# Fungal Communities Along a Small-Scale Elevational Gradient in an Alpine Tundra Are Determined by Soil Carbon Nitrogen Ratios

**DOI:** 10.3389/fmicb.2018.01815

**Published:** 2018-08-07

**Authors:** Yingying Ni, Teng Yang, Kaoping Zhang, Congcong Shen, Haiyan Chu

**Affiliations:** ^1^State Key Laboratory of Soil and Sustainable Agriculture, Institute of Soil Science, Chinese Academy of Sciences, Nanjing, China; ^2^University of Chinese Academy of Sciences, Beijing, China; ^3^State Key Laboratory of Urban and Regional Ecology, Research Center for Eco-Environmental Sciences, Chinese Academy of Sciences, Beijing, China

**Keywords:** soil fungal communities, functional guilds, C/N ratio, Illumina Miseq sequencing, elevational gradient, alpine tundra

## Abstract

Elevational gradients are associated not only with variations in temperature and precipitation, but also with shifts in vegetation types and changes in soil physicochemical properties. While large-scale elevational patterns of soil microbial diversity, such as monotonic declines and hump-shaped models, have been reported, it is unclear whether within-ecosystem elevational distribution patterns exist for soil fungal communities at the small scale. Using Illumina Miseq DNA sequencing, we present a comprehensive analysis of soil fungal diversity and community compositions in an alpine tundra ecosystem at elevations ranging from 2000 to 2500 m on the Changbai Mountain, China. Soil fungal community composition differed among elevations, and the fungal diversity (i.e., species richness and Chao1) increased along elevations. Soil fungal richness was negatively correlated with soil carbon/nitrogen (C/N) ratio, and community composition varied according to the C/N ratio. In addition, the relative abundances of Basidiomycota and Leotiomycetes were similarly negatively correlated with C/N ratio. For functional guilds, our data showed that mycoparasite and foliar epiphyte abundances were also influenced by C/N ratio. These results indicated that soil C/N ratio might be a key factor in determining soil fungal distribution at small-scale elevational gradients.

## Introduction

The study of elevational diversity patterns contributes to important insights for developing the general theory of species diversity (Lomolino, 2001). It also provides novel perspectives on the potential responses of biological communities and ecosystems to the changing environments (Sundqvist et al., 2013). While there is a long history of mapping the elevational distribution and abundance of macroorganisms across terrestrial ecosystems (Kessler et al., 2011; Descombes et al., 2017; Dvorsky et al., 2017; Forister et al., 2010), less is known about patterns of microbial elevational diversity. Some studies on bacterial diversity along elevational gradients have showed a range of microbial responses to increasing elevation, including decreasing (Bryant et al., 2008), unimodal (Singh et al., 2012), and inconsistent trends (Fierer et al., 2011; Shen et al., 2013; Xu et al., 2014; Wang et al., 2017). It has been demonstrated that bacterial diversity and community composition are affected by various factors, such as soil pH (Shen et al., 2013; Zhang et al., 2013), soil carbon and nitrogen content (Lin et al., 2015; Shen et al., 2015), and spatial distribution (Hayden and Beman, 2016), however, similar studies of soil fungi remain limited.

Soil is a vital habitat for morphologically and functionally diverse fungal groups, including decomposers, mutualists, and pathogens of plants and animals (Tedersoo et al., 2014, 2017). Fungal activity is known to influence the structures of plant and animal communities, as well as rates of ecosystem processes (Peay et al., 2016), but despite these critical roles in the ecosystem, soil fungal diversity, and biogeographic patterns remain generally under-studied and undefined (Pautasso, 2013). It is thought that drivers of soil fungal spatial distribution patterns vary according to ecosystem. For example, soil fungal communities were predominantly determined by the aboveground plant community in natural grasslands of Tibetan Plateau (Yang et al., 2017a), while in a high elevation desert, the fungal communities were determined by highly stochastic dispersal of fungal species (Yang et al., 2017b). In addition, a temperature was found to be the strongest predictor of soil fungal diversity in the maritime Antarctic (Newsham et al., 2016).

Altitude (elevation) has been shown to affect fungal communities, where, for example, lower phylogenetic diversity of alpine arbuscular mycorrhizal fungal communities were recorded at higher elevations in the North American Rocky Mountains (Egan et al., 2017). Drivers of soil fungal communities at varying elevations include climate variables: Bahram et al. (2012) found that monotonically declining species richness of ectomycorrhizal fungi with increasing elevation in temperate old-growth forests was constrained by the variation in mean annual precipitation and temperature. The effects of soil properties on elevational differences in microbial communities, however, are unclear. At a large-scale elevational mountain gradient, Shen et al. (2014) found that although soil eukaryotic microbial richness was not correlated with elevation, it was strongly correlated with soil pH on the Changbai Mountain, China. However, Ping et al. (2017) observed on the same mountain that soil fungal richness and evenness decreased with the increasing elevation gradient, and the community composition was significantly influenced by soil C/N ratio, moisture, pH, and total nitrogen (TN), suggesting that this elevational diversity pattern was scale dependent.

To date, most studies examining vertical fungal diversity patterns have focused on contrasting habitats and vegetation types over large-scale elevational gradients, with little attention to dynamics over smaller scales or within biomes (Guo et al., 2013; Sundqvist et al., 2013). Tundra biomes are under threat from climate change, since they are highly sensitive to the global warming, but the fungal communities in Changbai Mountain Tundra are little studied. Shi et al. (2015) found that the soil fungal community composition in an arctic tundra ecosystem was significantly affected by pH, ammonium concentration, C/N ratio, dissolved organic nitrogen (DON) content, and soil moisture content, however, it is not known whether these drivers of fungal community composition also apply in alpine tundra ecosystems.

The Changbai Natural Reserve, which encompasses within Changbai Mountain, is one of the largest biosphere reserves in China and has been spared from logging and other damaging human impacts (He et al., 2005). The vertical zonation of vegetation on Changbai Mountain transitions from broadleaf forest through to mixed, coniferous, and birch forests to alpine tundra (Dai et al., 2002). To increase our understanding of the structures of alpine tundra soil fungal communities, we investigated soil fungal diversity and community composition and soil physicochemical properties in the Changbai Mountain alpine tundra, along a small elevation gradient from 2000 to 2500 m. Specifically, we want to answer whether fungal diversity and community composition would change along the small-scale elevational gradient, and if so, what are the key drivers of the trend.

## Materials and Methods

### Study Site and Soil Sampling

The study site was located on Changbai Mountain (41°23′N–42°36′N, 126°55′E–129°00′E,) in northeast China, where alpine tundra occurs above 2000 m. The site and soil sampling methods have previously been fully described by Shen et al. (2013, 2015), so we provide a brief description here. On July 29, 2011, we selected alpine tundra study sites at six elevations between 2000 and 2500 m, separated by 100 m from the northern slope of Changbai Mountain. At each site, we created four 10 m × 10 m plots and from each of these, we randomly collected and combined six organic layer soil samples from an area of *c*. 10 cm × 10 cm and depth of 0–5 cm below the litter layer (*N* = 24). The fresh soil samples were sieved through a 2 mm sieve and visible roots and other residues were removed. Each of the 24 samples was divided into two subsamples, one was stored at -40°C for DNA extraction and the other was stored at 4°C for measurement of soil physicochemical properties. The main soil characteristics at the different elevations have been shown in the study of Shen et al. (2015).

### Soil Physicochemical Properties Analyses and Soil DNA Extraction

Soil pH, moisture, dissolved organic carbon (DOC), Nitrate (NO_3_^-^-N), ammonium (NH_4_^+^-N), DON, total carbon (TC), and TN were measured from each of the soil samples using methods described in detail by Shen et al. (2015). In brief, soil pH was measured after vigorous shaking water (1:5 w/v) supernatants for 30 min and soil moisture was measured gravimetrically. DOC was analyzed using a TOC analyzer (Analytik Jena, Multi N/C 3000, Germany) and NO_3_^-^-N, NH_4_^+^-N, and DTN were determined using continuous flow analytical system (Skalar, Holland). DON was calculated according the content of NO_3_^-^-N, NH_4_^+^-N, and DTN. Elemental analyzer (Vario MAX, Germany) was used to measure total TC and TN contents.

Soil DNA was extracted from 0.5 g fresh soil using a Fast DNA^®^ SPIN Kit for soil (MP Biomedicals, Santa Ana, CA, United States) according to the manufacturer’s instructions and crude DNA was purified by Power Clean^®^ Pro DNA Kit (MO BIO Laboratories, Inc.). The purified DNA was quantified by a Nano Drop ND-1000 spectrophotometer (Thermo Scientific, Wilmington, NC, United States) and then stored at -20°C for use.

### PCR Amplification and Illumina Miseq Sequencing

Primers ITS3F (5′-GCATCGATGAAGAACGCAGC-3′) and ITS4F (5′-TCCTCCGCTTATTGATATGC-3′) (Balajee et al., 2007) were used to amplify the ITS2 region. PCR was performed in 50 μl of reaction mixture containing 25 μl of Premix Taq DNA polymerase (TaKaRa, Japan), 0.5 μl of forward and reverse primers (20 μM), 23 μl of double distilled water (ddH_2_O), and 1 μl of DNA template (20 ng total soil DNA). The PCR cycling conditions were 94°C for 5 min, 32 cycles of 94°C for 30 s, 54°C for 30 s, and 72°C for 1 min. The PCR reaction was terminated at 72°C for 10 min and then cooled to 4°C. The PCR products were purified using EasyPure Quick Gel Extraction Kits (TransGen Biotech, Beijing, China) and quantified by the NanoDrop ND-1000 and then sequenced using the Illumina MiSeq PE 250 platform (Caporaso et al., 2012).

### Processing of Fungal ITS Sequencing Date

Sequences obtained by Illumina Miseq sequencing were processed and analyzed using the QIIME software package (version 1.9.0) following the default settings (Caporaso et al., 2010). Low quality sequences shorter than 200 bp were removed during filtering and chimera checking was performed using UCHIME algorithm (Edgar et al., 2011) by the USEARCH tool. Sequences were assigned to operational taxonomic units (OTUs) at 97% similarity, using the UCLUST algorithm (Edgar, 2010), which were used as measures of fungal species richness. Taxonomy was assigned to all representative sequences using the ribosomal database project (RDP) classifier based on the UNITE fungal ITS database (QIIME release, version 7.0) (Bengtsson-Palme et al., 2013; Nilsson et al., 2015). Singletons and non-fungal sequences were discarded and 39,900 sequences per sample were randomly subsampled to rarify the data sets to the same level for further analysis.

### Statistical Analyses

Alpha diversity was estimated using both taxonomic metrics (observed numbers of OTUs and Chao1 index). Chao1 was used to estimate the OTU richness based on frequencies of doubletons and singletons (Chao et al., 2009). Pearson’s correlation analysis in SPSS Statistics Version21 for Windows (IBM SPSS, United States) was used to test for correlations (*P* < 0.05) between fungal diversity and relative fungal abundance and elevation and soil physicochemical variable.

Principal Co-ordinates Analysis (PCoA) was used to compare beta diversity between samples along the elevations based on the Bray-Curtis distance matrix and visualized using the ggplot2 package in R statistical software (version 3.1.2). In addition, we tested for differences in community composition among the elevations using analysis of similarities (ANOSIM) (Clarke, 1993; Warton et al., 2012), based on Bray-Curtis distances, in the vegan package of R. Meanwhile, a permutational multivariate analysis of variance (PERMANOVA) was also used to determine the effects of elevation. We then analyzed beta diversity of fungi along the elevation gradient using non-metric multidimensional scaling (NMDS), based on Bray-Curtis distance, in the “vegan” package of R; NMDS analysis was performed with the metaMDS function (Oksanen et al., 2013).

We used distance-based multivariate linear model analysis (DistLM) (McArdle and Anderson, 2001) to assess the influence of the soil physicochemical variables on fungal community composition and marginal tests were used to assess the statistical significance and contribution of each variable separately. These tests were performed using the computer program DISTLM_forward3 (Anderson, 2003). Fungal functional guilds were assigned according to Tedersoo et al. (2014) and Nguyen et al. (2016a) using an open annotation tool (FUNGuild)^1^. Here, we only accepted the guild assignment that confidence ranking were “probable” and “highly probable,” which was recommended by Nguyen et al. (2016a). Fourteen fungal functional groups were detected including Ectomycorrhizal fungi, Plant pathogens, Mycoparasites, Wood saprotrophs, Dung saprotrophs, Animal pathogens, Ericoid mycorrhizal fungi, Lichenized, Lichenicolous, Foliar epiphytes, Arbuscular mycorrhizal fungi, Endophytes, Orchid mycorrhizal fungi, and Soil saprotrophs.

### Nucleotide Sequence Accession Numbers

Sequences were deposited in the NCBI Sequence Read Archive (SRA) under the accession number SRP110350^2^.

## Results

### Soil Physicochemical Properties

Soil physicochemical properties including soil pH, soil moisture, DOC, DON, NO_3_^-^-N, NH_4_^+^-N, TC, TN, and C/N ratio have been described in our previous study (Shen et al., 2015). Duncan’s multiple range tests showed that all the measured soil properties were significantly different between some elevations, except for pH (Shen et al., 2015). In addition, soil C/N ratio, the contents of TC and DOC were significantly negatively correlated with elevation (**Supplementary Table S1**).

### Fungal Diversity and Community Composition Along the Elevation Gradient

**FIGURE 1:**
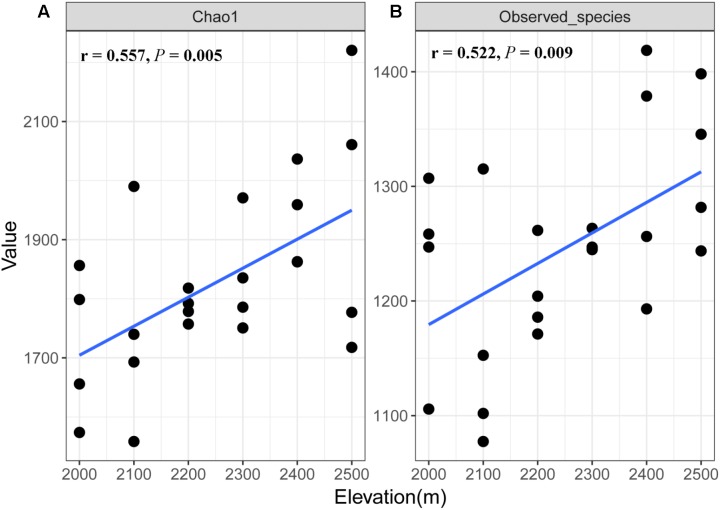
Relationship between fungal Chao1 **(A)** value and richness **(B)** and elevation.

A total of 1,173,608 quality sequences were obtained from the 24 soil samples, ranging from 39,905 to 60,636 per soil sample (average = 48,900). We found some differences in fungal richness between the different elevations (**Supplementary Table S2**), where species richness and Chao1 index of soil fungi increased with increasing elevation (*r* = 0.522, *P* = 0.009 and *r* = 0.557, *P* = 0.005, respectively; **Figure 1**).

**FIGURE 2:**
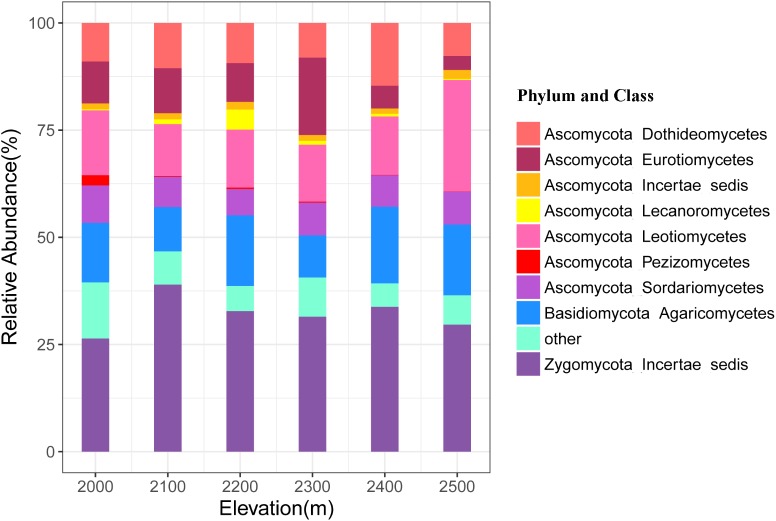
Relative abundances of the dominant fungal phyla and classes in soils at different elevations. The phylum and classes with relative abundances of <1% were assigned to “other.”

A total of 4493 unique OTUs were identified and assigned to six phyla across all soil samples at the six elevations. The Ascomycota, Zygomycota, and Basidiomycota were the dominant phyla, representing 52.34, 32.18, and 15.05% of the sequences, respectively, while relative abundance of the Chytridiomycota and Glomeromycota ranged from 0.05 to 0.17% and from 0.003 to 0.02%, respectively (**Figure 2** and **Supplementary Table S3**). Nine classes of fungi accounted for 91.97% of the total sequences, including Leotiomycetes, Dothideomycetes, Eurotiomycetes, Sordariomycetes, Ascomycota Incertae sedis, Lecanoromycetes, Pezizomycetes, Agaricomycetes, and Zygomycota Incertae_sedis (**Supplementary Table S3**). We allocated OTUs to 14 functional groups, among which, the most dominant were the ectomycorrhiza, plant pathogens, and mycoparasites with mean relative abundances >1% (**Supplementary Table S4**).

We found that while there was a difference in fungal community composition between 2500 m and the other elevations, there were no differences between 2000 and 2100 m, 2100 and 2200 m, 2200 and 2300 m, and 2300 and 2400 m using the ANOSIM test (**Table 1**). The first two axes of the PCoA explained 37.38% of the observed variation in community composition, and the biplot indicated that fungal communities tended to be more similar within than between elevations (**Supplementary Figure S1**). The significant difference in community composition between different elevations was also shown by PERMANOVA (*R*^2^ = 0.125, *P* = 0.001).

**Table 1:** Dissimilarities in fungal OTU community composition between elevations on Changbai Mountain as determined by ANOSIM.

**Elevation (m)**	**2100**	**2200**	**2300**	**2400**	**2500**
2000	0.29	0.11	**0.72**	**0.94**	**1.00**
2100		0.23	**0.91**	**0.69**	**1.00**
2200			0.2	0.3	**0.91**
2300				0.2	**1.00**
2400					**0.50**


*An R-value near +1 means that there is dissimilarity between the groups, while an. R-value near 0 indicates no significant dissimilarity between the groups. Values in. Bold indicate significant dissimilarity (*P* < 0.05).*

### Correlations Between Soil Variables and Fungal Diversity and Community Composition

**FIGURE 3:**
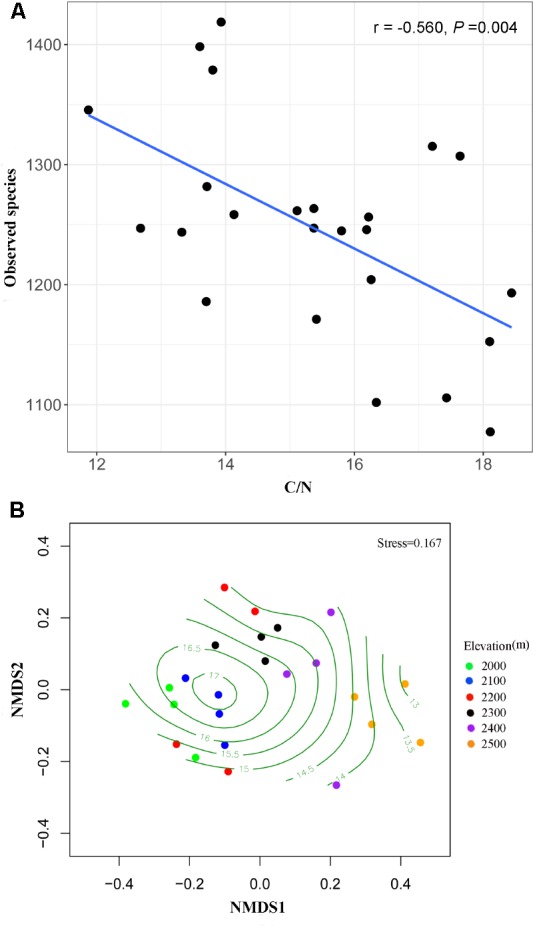
Effects of C/N ratio on the alpha and beta diversity of soil fungi. Alpha diversity index (observed species) decreased with increasing C/N ratio **(A)**. NMDS plot showing the best separation of samples along the C/N ratio gradients **(B)**.

Fungal species richness was negatively correlated with C/N ratio (*r* = -0.560, *P* = 0.004) (**Figure 3**) and TC (*r* = -0.410, *P* = 0.047), but was not associated with the other soil parameters (**Supplementary Table S5**). The distance-based multivariate showed that elevation, C/N ratio, TC, TN, and pH contributed to the variation in fungal community composition individually (**Table 2**). Among the soil physicochemical variables, C/N ratio provided the greatest explanatory power of the variation in community composition (11.2%) and it was selected as one of best environmental drivers in the multivariate model (**Table 2**). RDA analysis explained 36.35% of the total variation in soil fungal community composition, and the first two components explained 28.65% of the variation (**Supplementary Figure S2**). Soil C/N ratio was the strongest environmental driver of fungal community composition except elevation (**Table 2** and **Supplementary Figure S2**). The NMDS plot also exhibited the best separation of soil samples along the C/N ratio gradient (**Figure 3**).

**Table 2:** Distance-based multivariate linear model analysis (DistLM) for microbial fungal community composition.

Variable	% Var	Pseudo-*F*	*P*	Cum (%)
**Variables individually**				
Elevation	20.8	5.7784	**0.001**	
C/N	11.2	2.7701	**0.003**	
TC	9.3	2.2579	**0.010**	
ph	7.7	1.8408	**0.023**	
TN	8.0	1.9103	**0.033**	
DON	7.2	1.7024	0.053	
NO_3_^-^-N	6.2	1.4515	0.099	
DOC	6.4	1.4958	0.108	
NH_4_^+^-N	4.8	1.1016	0.315	
Moisture	2.6	0.5840	0.932	
**Variables fitted sequentially**				
Elevation	20.8	5.7784	**0.001**	20.8
C/N	5.7	1.6325	**0.031**	26.5
DON	5.7	1.6898	**0.036**	32.2
NH_4_^+^-N	5.2	1.5937	0.051	37.4
NO_3_^-^-N	4.5	1.4007	0.088	41.9
TN	4.0	1.2694	0.175	45.9
DOC	3.7	1.1803	0.246	49.6
Moisture	3.4	1.0926	0.334	53.0
pH	2.8	0.8813	0.630	55.8
TC	1.9	0.5787	0.901	57.7


The percentage of variance in species data explained by that variable is abbreviated as “% Var.” Values in boldface type indicate significant correlation (*P* < 0.05).

We found that the relative abundance of some fungal taxonomic groups, such as Basidiomycota, Ascomycota Incertae sedis, and Leotiomycetes, was significantly negatively correlated with soil C/N ratio (**Supplementary Tables S6, S7**). We also found a negative correlation between the relative abundance of Leotiomycetes and content of soil TN (*r* = -0.524, *P* = 0.009) and TC (*r* = -0.621, *P* = 0.001) (**Supplementary Table S7**). Among the functional guilds of fungi, the relative abundance of mycoparasites and foliar epiphytes were negatively correlated with soil C/N ratio (*r* = -0.405, *P* = 0.049 and *r* = -0.427, *P* = 0.037, respectively), and the relative abundance of foliar epiphytes was correlated with other soil properties, such as DOC, DON, TN, and TC (**Supplementary Table S8**).

## Discussion

Our results show that elevation strongly influenced the diversity of fungal communities in the tundra soils of Changbai Mountain, where diversity increased linearly with increasing elevation (**Figure 1**). Previous studies have observed different elevational patterns in soil fungal diversity. For example, Ping et al. (2017) found that soil fungal diversity decreased between the elevations from 699 to 937 m but increased between 937 and 1044 m in Korean pine forests on Changbai Mountain, showing a hollow curve’s pattern. Yang et al. (2017c) observed a monotonic decrease in soil fungal diversity along elevation from 700 to 2600 m by the culture-dependent method on Changbai Mountain. Our previous study showed that there was a lack of elevational pattern in soil fungal diversity at an altitudinal gradient from 530 to 2200 m on Changbai Mountain (Shen et al., 2014). The larger scale in elevational gradient contains different vegetation types, which is assumed to impact the elevational pattern in soil fungal diversity (He et al., 2017; Yang et al., 2017a). In addition, different microbial community analysis methods may also influence the observations on fungal distribution patterns along elevation.

To the best of our knowledge, our study is the first observation of a significant linear increase in soil fungal diversity along elevation in alpine tundra ecosystem. Similarly, a linear increase in diversity of foliar fungal endophytes along the increasing elevational gradient was observed within an Ermans birch forest on Changbai Mountain, which was strongly driven by leaf carbon content (Yang et al., 2016). Some environmental factors that differ among elevations may account for the diversity pattern along elevation. For example, soil pH has been found to drive the diversity pattern of soil fungi at the large-scale elevational gradient on Changbai Mountain (Shen et al., 2014), and climatic variables (temperature and precipitation) were assumed to be the potential drivers on the diversity pattern of ectomycorrhizal fungi along the elevational gradient in Hyrcanian forests of northern Iran (Bahram et al., 2012).

In this study, soil C/N ratio was significantly correlated with elevation and decreased with increasing elevations (*r* = -0.558, *P* = 0.005). Within the alpine tundra of Changbai Mountain, the plant communities at the lower elevations were dominated by dwarf shrubs (e.g., *Rhododendron aureum* and *Vaccinium uliginosum*), whereas the communities at the higher elevations were dominated by herbs/sedges, such as *Sanguisorba stipulata, Sanguisorba parviflora*, and *Carex atrata* (Wei et al., 2004; Zhang et al., 2017). Different dominant plant species at different elevations within alpine tundra may determine the variations of soil C/N ratio at the small-scale elevational gradient, which might be regulated by the components of root exudates and plant residues (Quideau et al., 2001; Knelman et al., 2012; Waring et al., 2015). In our study, soil fungal diversity significantly decreased with the increasing C/N ratio, and soil fungal community composition was mostly correlated with the C/N ratio among all soil variables. The above results indicated that C/N ratio was a key factor constraining both diversity pattern and community composition along elevation within alpine tundra on Changbai Mountain.

Similarly, the effect of C/N ratio on fungal diversity was also reported in other high and cold environments, such as the maritime Antarctic (Newsham et al., 2016) and the alpine grasslands of the Tibetan Plateau (Yang et al., 2017a). In addition, the effect of C/N ratio on soil fungal community composition was found in arctic tundra (Shi et al., 2015), permafrost-affected soils on the Tibetan Plateau (Zhang et al., 2014) and Korean pine forests on Changbai Mountain (Ping et al., 2017). Soil C/N ratio is generally thought as a proxy for nutrient availability (Cleveland and Liptzin, 2007) and as such, the change in ambient C/N ratio can substantially influence the fungal anabolism and foraging strategies (Prevost-Boure et al., 2011; Drake et al., 2013; Grosso et al., 2016). In terms of stoichiometry, the C/N ratio of a typical fungal biomass in soils was at 10–15 (Said et al., 2014), while the highest soil C/N ratio reached 18.44 in this study. High C/N ratio may broke the stoichiometric balance between soil and mycelia, restraining the activity of exoenzymes and accumulation of fungal biomass (Sinsabaugh et al., 2013). Of note, soil C/N ratio co-varied with the community composition of aboveground plants. On the one hand, rooting architectures and root exudates were quite different between dwarf shrubs and sedges/herbs, which dominated different elevations in this study. On the other hand, different dominant species at different elevations may select different fungal partners, given that mycorrhizal fungi is generally host specificity (Ishida et al., 2007; Tedersoo et al., 2008). Consequently, the observed pattern driven by C/N ratio in this study may be partly attributed to the variation of aboveground plant communities. Previously, many studies reported that plant species identity strongly affected soil fungal distributions (Tedersoo et al., 2016; Dassen et al., 2017; Gao et al., 2017). Nonetheless, the relative effects of plant dominant species and soil C/N ratio on soil fungal communities within alpine tundra still warrant further investigations.

In this study, the phylum of Zygomycota was more dominant than Basidiomycota, which was different from the observations in other tundra studies (Nemergut et al., 2005; Timling et al., 2014). The fungi in Zygomycota are generally regarded as the degraders of sucrose and cellulose, while most fungi in Basidiomycota are thought as lignin-degrading fungi (Waksman et al., 1928). We also found that ectomycorrhizal fungi, plant pathogens, and mycoparasites were the dominant functional guilds. The dominant plant species (e.g., dwarf shrubs and sedges) in alpine tundra were the host of ectomycorrhizal fungi (Wang and Qiu, 2006), which provided lots of living space for ectomycorrhizal fungi. The ectomycorrhizal fungi and plant pathogens have also been found as the dominant functional guilds in the arctic (Timling et al., 2014). Here, we also found that different taxonomic groups and functional guilds responded to different environmental variables (**Supplementary Tables S6, S7, S8**). For example, the relative abundance of Ascomycota was significantly correlated with DON, while the relative abundance of Basidiomycota was significantly correlated with soil C/N ratio. The relative abundance of ectomycorrhizal fungi was mostly related with NH_4_^+^-N, while the mycoparasite and foliar epiphytes were mostly related with soil C/N ratio. In a field experiment, Nguyen et al. (2016b) found that the compositions of ectomycorrhizal fungi and saprotrophs were affected by host plant phylogenetic diversity and plant community structure, respectively. Vasutova et al. (2017) observed tree height was an important driver for the mycorrhizal fungi, whereas, the community composition of saprotrophs were mainly shaped by vegetation and edaphic variables. Solly et al. (2017) revealed that the relative abundance of ectomycorrhizal fungi was positively correlated with NH_4_^+^ concentrations but negatively correlated with soil C/N ratio in an alpine treeline. The responses of ectomycorrhizal fungal communities to some environmental factors under warming conditions may lead to major shifts in dominant taxa and mycelia production (Peter et al., 2001; Lilleskov et al., 2002; Solly et al., 2017). Therefore, in the context of climate changing, the studies on the shifts of functional guilds in alpine tundra should be more cautious.

## Conclusion

In summary, soil fungal community composition and diversity changed along a small-scale elevational gradient in an alpine tundra on Changbai Mountain. Soil C/N ratio was not only negatively correlated with fungal diversity, but also significantly correlated with fungal community composition, and the relative abundances of specific phyla, class, and functional guilds. The results suggest that soil C/N ratio is a key factor for determining fungal elevational distribution pattern in alpine tundra ecosystems.

## Author Contributions

HC designed the study. CS collected soil samples. YN and CS conducted the experiment. YN, TY, and KZ analyzed the data. YN, TY, and HC wrote the manuscript.

## Conflict of Interest Statement

The authors declare that the research was conducted in the absence of any commercial or financial relationships that could be construed as a potential conflict of interest.

## Footnotes

1 http://www.stbates.org/guilds/app.php

2 https://www.ncbi.nlm.nih.gov/sra/?term=SRP110350

## References

[B1] Anderson M. J. (2003). *DISTLM forward: a FORTRAN Computer Program to Calculate a Distance-Based Multivariate Analysis for a Linear Model Using Forward Selection.* Auckland: Department of Statistics, University of Auckland.21988714

[B2] Bahram M. Polme S. Koljalg U. Zarre S. Tedersoo L. (2012). Regional and local patterns of ectomycorrhizal fungal diversity and community structure along an altitudinal gradient in the Hyrcanian forests of northern Iran. *New Phytol.* 193 465–473. 10.1111/j.1469-8137.2011.03927.x 21988714

[B3] Balajee S. A. Sigler L. Brandt M. E. (2007). DNA and the classical way: identification of medically important molds in the 21st century. *Med. Mycol.* 45 475-490. 10.1080/13693780701449425 17710617

[B4] Bengtsson-Palme J. Ryberg M. Hartmann M. Branco S. Wang Z. Godhe A. (2013). Improved software detection and extraction of ITS1 and ITS2 from ribosomal ITS sequences of fungi and other eukaryotes for analysis of environmental sequencing data. *Methods Ecol. Evol.* 4 914–919. 10.1111/2041-210X.12073

[B5] Bryant J. A. Lamanna C. Morlon H. Kerkhoff A. J. Enquist B. J. Green J. L. (2008). Microbes on mountainsides: contrasting elevational patterns of bacterial and plant diversity. *Proc. Natl. Acad. Sci. U.S.A.* 105 11505–11511. 10.1073/pnas.0801920105 18695215 PMC2556412

[B6] Caporaso J. G. Kuczynski J. Stombaugh J. Bittinger K. Bushman F. D. Costello E. K. (2010). QIIME allows analysis of high-throughput community sequencing data. *Nat. Methods* 7 335–336. 10.1038/nmeth.f.303 20383131 PMC3156573

[B7] Caporaso J. G. Lauber C. L. Walters W. A. Berg-Lyons D. Huntley J. Fierer N. (2012). Ultra-high-throughput microbial community analysis on the Illumina HiSeq and MiSeq platforms. *ISME J.* 6 1621–1624. 10.1038/ismej.2012.8 22402401 PMC3400413

[B8] Chao A. Colwell R. K. Lin C. W. Gotelli N. J. (2009). Sufficient sampling for asymptotic minimum species richness estimators. *Ecology* 90 1125–1133. 10.1890/07-2147.1 19449706

[B9] Clarke K. R. (1993). Non-parametric multivariate analyses of changes in community structure. *Aust. J. Ecol.* 18 117–143. 10.1111/j.1442-9993.1993.tb00438.x

[B10] Cleveland C. C. Liptzin D. (2007). C: N: P stoichiometry in soil: is there a Redfield ratio for the microbial biomass? *Biogeochemistry* 85 235–252. 10.1007/s10533-007-9132-0

[B11] Dai L. M. Wu G. Zhao J. Z. Kong H. M. Shao G. F. Deng H. B. (2002). Carbon cycling of alpine tundra ecosystems on Changbai Mountain and its comparison with arctic tundra. *Sci. China Ser. D* 45 903–910. 10.1360/02yd9089

[B12] Dassen S. Cortois R. Martens H. de Hollander M. Kowalchuk G. A. van der Putten W. H. (2017). Differential responses of soil bacteria, fungi, archaea and protists to plant species richness and plant functional group identity. *Mol. Ecol.* 26 4085–4098. 10.1111/mec.14175 28489329

[B13] Descombes P. Marchon J. Pradervand J. Bilat J. Guisan A. Rasmann S. (2017). Community-level plant palatability increases with elevation as insect herbivore abundance declines. *J. Ecol.* 105 142–151. 10.1111/1365-2745.12664

[B14] Drake J. E. Darby B. A. Giasson M. A. Kramer M. A. Phillips R. P. Finzi A. C. (2013). Stoichiometry constrains microbial response to root exudation-insights from a model and a field experiment in a temperate forest. *Biogeosciences* 10 821–838. 10.5194/bg-10-821-2013

[B15] Dvorsky M. Macek M. Kopecky M. Wild J. Dolezal J. (2017). Niche asymmetry of vascular plants increases with elevation. *J. Biogeogr.* 44 1418–1425. 10.1111/jbi.13001

[B16] Edgar R. C. (2010). Search and clustering orders of magnitude faster than BLAST. *Bioinformatics* 26 2460–2461. 10.1093/bioinformatics/btq461 20709691

[B17] Edgar R. C. Haas B. J. Clemente J. C. Quince C. Knight R. (2011). UCHIME improves sensitivity and speed of chimera detection. *Bioinformatics* 27 2194–2200. 10.1093/bioinformatics/btr381 21700674 PMC3150044

[B18] Egan C. P. Callaway R. M. Hart M. M. Pither J. Klironomos J. (2017). Phylogenetic structure of arbuscular mycorrhizal fungal communities along an elevation gradient. *Mycorrhiza* 27 273–282. 10.1007/s00572-016-0752-x 27909817

[B19] Fierer N. McCain C. M. Meir P. Zimmermann M. Rapp J. M. Silman M. R. (2011). Microbes do not follow the elevational diversity patterns of plants and animals. *Ecology* 92 797–804. 10.1890/10-1170.1 21661542

[B20] Forister M. L. McCall A. C. Sanders N. J. Fordyce J. A. Thorne J. H. O’Brien J. (2010). Compounded effects of climate change and habitat alteration shift patterns of butterfly diversity. *Proc. Natl. Acad. Sci. U.S.A.* 107 2088–2092. 10.1073/pnas.0909686107 20133854 PMC2836664

[B21] Gao C. Shi N. N. Chen L. Ji N. N. Wu B. W. Wang Y. L. (2017). Relationships between soil fungal and woody plant assemblages differ between ridge and valley habitats in a subtropical mountain forest. *New Phytol.* 213 1874–1885. 10.1111/nph.14287 28164340

[B22] Grosso F. Baath E. De Nicola F. (2016). Bacterial and fungal growth on different plant litter in Mediterranean soils: Effects of C/N ratio and soil pH. *Appl. Soil Ecol.* 108 1–7. 10.1016/j.apsoil.2016.07.020

[B23] Guo Q. F. Kelt D. A. Sun Z. Y. Liu H. X. Hu L. J. Ren H. (2013). Global variation in elevational diversity patterns. *Sci. Rep.* 3:3007. 10.1038/srep03007 24157658 PMC6505670

[B24] Hayden C. J. Beman J. M. (2016). Microbial diversity and community structure along a lake elevation gradient in Yosemite National Park, California, USA. *Environ. Microbiol.* 18 1782–1791. 10.1111/1462-2920.12938 26058326

[B25] He H. S. Hao Z. Q. Mladenoff D. J. Shao G. F. Hu Y. M. Chang Y. (2005). Simulating forest ecosystem response to climate warming incorporating spatial effects in north-eastern China. *J. Biogeogr.* 32 2043–2056. 10.1111/j.1365-2699.2005.01353.x

[B26] He J. H. Tedersoo L. H. Hu A. Han C. H. He D. Wei H. (2017). Greater diversity of soil fungal communities and distinguishable seasonal variation in temperate deciduous forests compared with subtropical evergreen forests of eastern China. *FEMS Microbiol. Ecol.* 93:7. 10.1093/femsec/fix069 28854678

[B27] Ishida T. A. Nara K. Hogetsu T. (2007). Host effects on ectomycorrhizal fungal communities: insight from eight host species in mixed conifer-broadleaf forests. *New Phytol.* 174 430–440. 10.1111/j.1469-8137.2007.02016.x 17388905

[B28] Kessler M. Kluge J. Hemp A. Ohlemuller R. (2011). A global comparative analysis of elevational species richness patterns of ferns. *Global Ecol. Biogeogr.* 20 868–880. 10.1111/j.1466-8238.2011.00653.x

[B29] Knelman J. E. Legg T. M. O’Neill S. P. Washenberger C. L. González A. Cleveland C. C. (2012). Bacterial community structure and function change in association with colonizer plants during early primary succession in a glacier forefield. *Soil Biol. Biochem.* 46 172–180. 10.1016/j.soilbio.2011.12.001

[B30] Lilleskov E. A. Hobbie E. A. Fahey T. J. (2002). Ectomycorrhizal fungal taxa differing in response to nitrogen deposition also differ in pure culture organic nitrogen use and natural abundance of nitrogen isotopes. *New Phytol.* 154 219–231. 10.1046/j.1469-8137.2002.00367.x

[B31] Lin Y. T. Whitman W. B. Coleman D. C. Shi S. Y. Tang S. L. Chiu C. Y. (2015). Changes of soil bacterial communities in bamboo plantations at different elevations. *FEMS Microbiol. Ecol.* 915. 10.1093/femsec/fiv033 25873459

[B32] Lomolino M. V. (2001). Elevation gradients of species-density, historical and prospective views. *Global Ecol. Biogeogr.* 10 3–13. 10.1046/j.1466-822x.2001.00229.x

[B33] McArdle B. H. Anderson M. J. (2001). Fitting multivariate models to community data: a comment on distance-based redundancy analysis. *Ecology* 82 290–297. 10.1890/0012-9658(2001)082[0290:FMMTCD]2.0.CO;2

[B34] Nemergut D. R. Costello E. K. Meyer A. F. Pescador M. Y. Weintraub M. N. Schmidt S. K. (2005). Structure and function of alpine and arctic soil microbial communities. *Res. Microbiol.* 156 775–784. 10.1016/j.resmic.2005.03.004 15922566

[B35] Newsham K. K. Hopkins D. W. Carvalhais L. C. Fretwell P. T. Rushton S. P. O’Donnell A. G. (2016). Relationship between soil fungal diversity and temperature in the maritime Antarctic. *Nat. Clim. Change* 6 182–186. 10.1038/nclimate2806

[B36] Nguyen N. H. Song Z. W. Bates S. T. Branco S. Tedersoo L. Menke J. (2016a). FUNGuild: an open annotation tool for parsing fungal community datasets by ecological guild. *Fungal Ecol.* 20 241–248. 10.1016/j.funeco.2015.06.006

[B37] Nguyen N. H. Williams L. J. Vincent J. B. Stefanski A. Cavender-Bares J. Messier C. (2016b). Ectomycorrhizal fungal diversity and saprotrophic fungal diversity are linked to different tree community attributes in a field-based tree experiment. *Mol. Ecol.* 25 4032–4046. 10.1111/mec.13719 27284759

[B38] Nilsson R. H. Tedersoo L. Ryberg M. Kristiansson E. Hartmann M. Unterseher M. (2015). A comprehensive, automatically updated fungal ITS sequence dataset for reference-based chimera control in environmental sequencing efforts. *Microbes Environ.* 30 145–150. 10.1264/jsme2.ME14121 25786896 PMC4462924

[B39] Oksanen J. Blanchet F. G. Kindt R. Legendre P. Michin P. R. O’Hara R. B. (2013). *Vegan: Community Ecology Package 2.0-10.* Available at: http://CRAN.R-project.org/package=vegan

[B40] Pautasso M. (2013). Fungal under-representation is (slowly) diminishing in the life sciences. *Fungal Ecol.* 6 129–135. 10.1016/j.funeco.2012.04.004

[B41] Peay K. G. Kennedy P. G. Talbot J. M. (2016). Dimensions of biodiversity in the Earth mycobiome. *Nat. Rev. Microbial.* 14 434–447. 10.1038/nrmicro.2016.59 27296482

[B42] Peter M. Ayer F. Egli S. (2001). Nitrogen addition in a Norway spruce stand altered macromycete sporocarp production and below-ground ectomycorrhizal species composition. *New Phytol.* 149 311–325. 10.1046/j.1469-8137.2001.00030.x 33874626

[B43] Ping Y. Han D. X. Wang N. Hu Y. B. Mu L. Q. Feng F. J. (2017). Vertical zonation of soil fungal community structure in a Korean pine forest on Changbai Mountain, China. *World J. Microbiol. Biotechnol.* 33:12. 10.1007/s11274-016-2133-1 27885566

[B44] Prevost-Boure N. C. Christen R. Dequiedt S. Mougel C. Lelievre M. Jolivet C. (2011). Validation and application of a PCR primer set to quantify fungal communities in the soil environment by real-time quantitative PCR. *PLoS One* 6:e24166. 10.1371/journal.pone.0024166 21931659 PMC3169588

[B45] Quideau S. A. Chadwick O. A. Benesi A. Graham R. C. Anderson M. A. (2001). A direct link between forest vegetation type and soil organic matter composition. *Geoderma* 104 41–60. 10.1016/S0016-7061(01)00055-6

[B46] Said F. M. Brooks J. Chisti Y. (2014). Optimal C:N ratio for the production of red pigments by Monascus ruber. *World J. Microb. Biotech.* 30 2471–2479. 10.1007/s11274-014-1672-6 24845168

[B47] Shen C. C. Liang W. J. Shi Y. Lin X. G. Zhang H. Y. Wu X. (2014). Contrasting elevational diversity patterns between eukaryotic soil microbes and plants. *Ecology* 95 3190–3202. 10.1890/14-0310.1

[B48] Shen C. C. Ni Y. Y. Liang W. J. Wang J. J. Chu H. Y. (2015). Distinct soil bacterial communities along a small-scale elevational gradient in alpine tundra. *Front. Microbiol.* 6:582. 10.3389/fmicb.2015.00582 26217308 PMC4493907

[B49] Shen C. C. Xiong J. B. Zhang H. Y. Feng Y. Z. Lin X. G. Li X. Y. (2013). Soil pH drives the spatial distribution of bacterial communities along elevation on Changbai Mountain. *Soil Biol. Biochem.* 57 204–211. 10.1016/j.soilbio.2012.07.013

[B50] Shi Y. Xiang X. Shen C. C. Chu H. Y. Neufeld J. D. Walker V. K. (2015). Vegetation-associated impacts on arctic tundra bacterial and microeukaryotic communities. *Appl. Envrion. Microb.* 81 492–501. 10.1128/AEM.03229-14 25362064 PMC4277566

[B51] Singh D. Takahashi K. Kim M. Chun J. Adams J. M. (2012). A Hump-Backed trend in bacterial diversity with elevation on Mount Fuji, Japan. *Microb. Ecol.* 63 429–437. 10.1007/s00248-011-9900-1 21735154

[B52] Sinsabaugh R. L. Manzoni S. Moorhead D. L. Richter A. (2013). Carbon use efficiency of microbial communities: stoichiometry, methodology and modelling. *Ecol. Lett.* 16 930–939. 10.1111/ele.12113 23627730

[B53] Solly E. F. Lindahl B. D. Dawes M. A. Peter M. Souza R. C. Rixen C. (2017). Experimental soil warming shifts the fungal community composition at the alpine treeline. *New Phytol.* 215 766–778. 10.1111/nph.14603 28543616

[B54] Sundqvist M. K. Sanders N. J. Wardle D. A. (2013). Community and ecosystem responses to elevational gradients: processes, mechanisms, and insights for global change. *Annu. Rev. Ecol. Evol. Syst.* 44 261–280. 10.1146/annurev-ecolsys-110512-135750

[B55] Tedersoo L. Bahram M. Cajthaml T. Põlme S. Hiiesalu I. Anslan S. (2016). Tree diversity and species identity effects on soil fungi, protists and animals are context dependent. *ISME J.* 10 346–362. 10.1038/ismej.2015.116 26172210 PMC4737927

[B56] Tedersoo L. Bahram M. Polme S. Koljalg U. Yorou N. S. Wijesundera R. (2014). Fungal biogeography: Global diversity and geography of soil fungi. *Science* 346:1256688. 10.1126/science.1256688 25430773

[B57] Tedersoo L. Bahram M. Puusepp R. Nilsson R. H. James T. Y. (2017). Novel soil-inhabiting clades fill gaps in the fungal tree of life. *Microbiome* 5:42. 10.1186/s40168-017-0259-5 28388929 PMC5385062

[B58] Tedersoo L. Jairus T. Horton B. M. Abarenkov K. Suvi T. Saar I. (2008). Strong host preference of ectomycorrhizal fungi in a Tasmanian wet sclerophyll forest as revealed by DNA barcoding and taxon-specific primers. *New Phytol.* 180 479–490. 10.1111/j.1469-8137.2008.02561.x 18631297

[B59] Timling I. Walker D. A. Nusbaum C. Lennon N. J. Taylor D. L. (2014). Rich and cold: Diversity, distribution and drivers of fungal communities in patterned-ground ecosystems of the North American Arctic. *Mol. Ecol.* 23 3258–3272. 10.1111/mec.12743 24689939

[B60] Vasutova M. Edwards-Jonasova M. Baldrian P. Cermak M. Cudlin P. (2017). Distinct environmental variables drive the community composition of mycorrhizal and saprotrophic fungi at the alpine treeline ecotone. *Fungal Ecol.* 27 116–124. 10.1016/j.funeco.2016.08.010

[B61] Waksman S. A. Tenney F. G. Stevens K. R. (1928). The role of microorganisms in the transformation of organic matter in forest soils. *Ecology* 9 126–144. 10.2307/1929350

[B62] Wang B. Qiu Y. L. (2006). Phylogenetic distribution and evolution of mycorrhizas in land plants. *Mycorrhiza* 16 299–363. 10.1007/s00572-005-0033-6 16845554

[B63] Wang J. Meier S. Soininen J. Casamayor E. O. Pan F. Tang X. (2017). Regional and global elevational patterns of microbial species richness and evenness. *Ecography* 40 393–402. 10.1111/ecog.02216

[B64] Waring B. G. Alvarez-Cansino L. Barry K. E. Becklund K. K. Dale S. Gei M. G. (2015). Pervasive and strong effects of plants on soil chemistry: a meta-analysis of individual plant ‘Zinke’ effects. *Proc. R. Soc. B-Biol. Sci.* 282 91–98. 10.1098/rspb.2015.1001 26224711 PMC4528518

[B65] Warton D. I. Wright S. T. Wang Y. (2012). Distance-based multivariate analyses confound location and dispersion effects. *Methods Ecol. Evol.* 3 89–101. 10.1111/j.2041-210X.2011.00127.x

[B66] Wei J. Wu G. Deng H. B. (2004). Vegetation biomass distribution characteristics of alpine tundra ecosystem in Changbai Mountains. *Chin. J. Appl. Ecol.* 15 1999–2004. 15707302

[B67] Xu M. Li X. L. Cai X. B. Gai J. P. Li X. L. Christie P. (2014). Soil microbial community structure and activity along a montane elevational gradient on the Tibetan Plateau. *Eur. J. Soil Biol.* 64 6–14. 10.1016/j.ejsobi.2014.06.002

[B68] Yang H. Lu G. Z. Jiang H. M. Shi D. N. Liu Z. H. (2017c). Diversity and distribution of soil micro-fungi along an elevation gradient on the north slope of Changbai Mountain. *J. For. Res.* 28 831–839. 10.1007/s11676-016-0344-9

[B69] Yang T. Adams J. M. Shi Y. He J. S. Jing X. Chen L. T. (2017a). Soil fungal diversity in natural grasslands of the Tibetan Plateau: associations with plant diversity and productivity. *New Phytol.* 215 756–765. 10.1111/nph.14606 28542845

[B70] Yang T. Adams J. M. Shi Y. Sun H. B. Cheng L. Zhang Y. J. (2017b). Fungal community assemblages in a high elevation desert environment: absence of dispersal limitation and edaphic effects in surface soil. *Soil Biol. Biochem.* 115 393–402. 10.1016/j.soilbio.2017.09.013

[B71] Yang T. Weisenhorn P. Gilbert J. A. Ni Y. Y. Sun R. B. Shi Y. (2016). Carbon constrains fungal endophyte assemblages along the timberline. *Environ. Microbiol.* 18 2455–2469. 10.1111/1462-2920.13153 26627043

[B72] Zhang B. Liang C. He H. Zhang X. (2013). Variations in soil microbial communities and residues along an altitude gradient on the northern slope of Changbai Mountain, China. *PLoS One* 8:e66184. 10.1371/journal.pone.0066184 23776630 PMC3679006

[B73] Zhang X. F. Xu S. J. Li C. M. Zhao L. Feng H. Y. Yue G. Y (2014). The soil carbon/nitrogen ratio and moisture affect microbial community structures in alkaline permafrost-affected soils with different vegetation types on the *Tibetan plateau*. *Res. Microbiol.* 165 128–139. 10.1016/j.resmic.2014.01.002 24463013

[B74] Zhang Y. J. Jin Y. H Gu X. N. Xu J. W Tao Y. He H. S. (2017). Vegetation change in relation to soil microbes, enzyme activity and soil fertility in the tundra of Changbai Mountain. *Chin. J. Ecol.* 36 3086–3093.

