# Supplementary material for: Fungal Communities Along a Small-Scale Elevational Gradient in an Alpine Tundra Are Determined by Soil Carbon Nitrogen Ratios
Source: Front Microbiol. 2018 Aug 7;9:1815. doi: 10.3389/fmicb.2018.01815 (PMC6091257; doi:10.3389/fmicb.2018.01815)
Supplement: Supplementary file 1 [file Data_Sheet_1.pdf]

Supplementary materials

Table S1 Pearson correlations (r) between tundra soil characteristics and the elevations.

	Elevation	
	r	*P*
C/N ratio	**−0.558****	**0.005**
TC (%)	**−0.485***	**0.016**
DOC ($mg\cdot kg^{-1}$)	**−0.470***	**0.021**
NO_3^+-N ($mg\cdot kg^{-1}$)	0.402	0.052
TN (%)	−0.4	0.052
pH	0.362	0.082
DON ($mg\cdot kg^{-1}$)	−0.31	0.140
NH_4^+-N ($mg\cdot kg^{-1}$)	0.157	0.465
Moisture (%)	0.064	0.766

Values in bold indicate significant correlations (*$P < 0.05$, **$P < 0.01$).

Table S2 Alpha diversity of fungal communities at different elevations.

	2000m	2100m	2200m	2300m	2400m	2500m
observed species	1230±87ab	1162±107a	1206±40ab	1250±9ab	1312±105b	1317±68b
Chao1	1721±129a	1745±180a	1786±25a	1835±97a	1930±84a	1944±237a

Different letters indicate statistical differences along the elevations using Duncan Multiple Range Test for comparisons.

Table S3 Relative abundances (%) of classes of fungi at different elevations.

Taxon	Total	2000m	2100m	2200m	2300m	2400m	2500m
Ascomycota							
Leotiomycetes	15.62	15.13	12.12	13.50	13.28	13.64	26.02
Dothideomycetes	9.89	9.00	10.56	9.38	8.09	14.64	7.69
Eurotiomycetes	9.32	9.77	10.49	9.03	18.05	5.32	3.27
Sordariomycetes	7.44	8.74	7.08	6.16	7.65	7.37	7.62
Ascomycota; Other	6.41	11.94	6.81	5.14	8.10	3.90	2.59
Incertae sedis	1.54	1.36	1.44	1.81	1.35	1.22	2.07
Lecanoromycetes	1.30	0.25	1.11	4.65	0.91	0.62	0.27
Pezizomycetes	0.53	2.37	0.14	0.33	0.21	0.05	0.06
Geoglossomycetes	0.23	0.25	0.01	0.07	0.09	0.40	0.58
Orbiliomycetes	0.05	0.06	0.05	0.03	0.04	0.05	0.03
Saccharomycetes	0.01	0.01	0.00	0.00	0.00	0.01	0.01
Archaeorhizomycetes	0.00	0.00	0.00	0.00	0.00	0.00	0.00
Basidiomycota							
Agaricomycetes	14.15	13.88	10.33	16.46	9.82	17.86	16.53
Tremellomycetes	0.74	0.30	0.39	0.36	0.30	0.34	2.73
Microbotryomycetes	0.09	0.05	0.03	0.07	0.06	0.07	0.25
Basidiomycota; Other	0.05	0.07	0.05	0.02	0.08	0.05	0.05
Pucciniomycetes	0.01	0.01	0.00	0.01	0.01	0.01	0.01
Agaricostilbomycetes	0.01	0.00	0.00	0.00	0.03	0.01	0.00
Exobasidiomycetes	0.01	0.01	0.00	0.00	0.01	0.00	0.01
Ustilaginomycetes	0.00	0.00	0.00	0.00	0.00	0.00	0.00
Atractiellomycetes	0.00	0.00	0.00	0.00	0.00	0.00	0.00
Cystobasidiomycetes	0.00	0.00	0.00	0.00	0.00	0.00	0.00
Wallemiomycetes	0.00	0.00	0.00	0.00	0.00	0.00	0.00
Incertae sedis	0.00	0.00	0.00	0.00	0.00	0.00	0.00
Zygomycota							
Incertae sedis	32.18	26.40	38.98	32.78	31.50	33.80	29.64
Chytridiomycota							
Chytridiomycetes	0.08	0.04	0.05	0.05	0.17	0.11	0.10
Chytridiomycota; Other	0.00	0.01	0.00	0.00	0.00	0.00	0.01
Glomeromycota							
Glomeromycetes	0.01	0.02	0.00	0.00	0.01	0.02	0.01
Rozellomycota							
unidentified	0.00	0.00	0.00	0.00	0.00	0.00	0.00
Fungi; Other	0.33	0.31	0.34	0.13	0.24	0.49	0.45

Table S4 Relative abundances (%) of functional groups at different elevations.

Functional Group	Total	2000m	2100m	2200m	2300m	2400m	2500m
Undefined saprotrophs	43.353	43.758	48.315	45.398	40.964	43.507	38.179
Unassigned	40.436	42.873	40.635	35.795	46.054	32.347	44.910
Ectomycorrhizal	7.465	6.174	3.000	12.221	4.080	11.577	7.737
Plant pathogens	3.893	2.872	3.298	3.145	3.826	7.201	3.014
Undefined endophytes	2.097	1.766	2.572	1.489	1.768	1.899	3.088
Mycoparasites	1.188	0.645	0.596	0.622	2.103	2.065	1.094
Wood saprotrophs	0.525	0.735	0.617	0.299	0.328	0.341	0.830
Dung Saprotroph	0.481	0.518	0.440	0.491	0.521	0.465	0.449
Animal pathogens	0.273	0.202	0.219	0.204	0.186	0.372	0.454
Ericoid mycorrhizal	0.166	0.402	0.160	0.122	0.058	0.106	0.148
Lichenized	0.072	0.016	0.128	0.093	0.085	0.079	0.031
Lichenicolous	0.019	0.000	0.000	0.102	0.001	0.001	0.011
Foliar Epiphyte	0.013	0.008	0.006	0.008	0.008	0.014	0.036
Arbuscular mycorrhizal	0.009	0.018	0.003	0.003	0.012	0.014	0.007
Endophyte	0.005	0.007	0.005	0.003	0.002	0.008	0.007
Orchid Mycorrhizal	0.004	0.006	0.001	0.004	0.004	0.004	0.001
Soil Saprotroph	0.002	0.000	0.004	0.001	0.002	0.001	0.003

Table S5 Pearson correlations (r) between fungal diversity and soil characteristics.

	pH	Moisture	DOC	NO_3^+-	NH_4^+-	DON	TN	TC	C/N ratio
observed species	0.153	-0.130	0.059	0.213	0.330	-0.026	-0.256	**-0.410***	**-0.560****
Chao1	0.100	0.021	0.034	0.254	0.189	0.002	-0.181	-0.26	-0.335

Values in bold indicate significant correlations (*$P < 0.05$, **$P < 0.01$).

Table S6 Correlation between relative abundance of dominant fungi phyla and soil physicochemical variables along the elevational gradients.

Phylum	pH	Moisture	DOC	NO_3^+-N	NH_4^+-N	DON	TN	TC	C/N ratio
Ascomycota	0.077	-0.163	0.26	-0.118	-0.023	**0.424***	0.144	0.088	-0.047
Basidiomycota	0.188	-0.109	-0.334	-0.101	0.275	-0.122	-0.141	-0.274	**-0.425***
Zygomycota	-0.181	0.211	-0.033	0.161	-0.144	-0.303	-0.039	0.089	0.296

Values in bold indicate significant correlations (*$P < 0.05$, **$P < 0.01$).

Table S7 Correlation between relative abundance of dominant classes of fungi and soil physicochemical variables along the elevational gradients.

Class	Elevation	pH	Moisture	DOC	NO_3^--N	NH_4^+-N	DON	TN	TC	C/N ratio
Ascomycota_Dothideomycetes	0.057	-0.124	**0.417***	0.31	**0.5***	0.319	0.182	0.203	0.235	0.178
Ascomycota_Eurotiomycetes	-0.311	0.155	-0.135	0.113	-0.395	-0.321	0.182	0.2	0.267	0.338
Ascomycota_Incertae sedis	0.212	-0.156	-0.176	-0.136	0.097	0.073	0.034	-0.203	-0.33	**-0.482***
Ascomycota_Leotiomycetes	**0.480***	0.21	-0.248	-0.298	0.065	-0.019	-0.324	**-0.524****	**-0.621****	**-0.614****
Ascomycota_Sordariomycetes	-0.084	-0.093	**-0.477***	-0.221	**-0.463***	0.019	-0.153	-0.265	-0.363	-0.397
Ascomycota_Lecanoromycetes	-0.082	-0.038	0.079	0.198	0.128	0.227	**.601****	**.436***	0.333	0.001
Ascomycota_Pezizomycetes	-0.368	**-.545****	-0.019	0.042	0.154	0.33	0.004	0.044	-0.033	-0.134
Basidiomycota_Agaricomycetes	0.23	0.17	-0.098	-0.277	-0.162	0.296	-0.061	-0.034	-0.163	-0.341
Zygomycota_Incertae sedis	-0.003	-0.181	0.211	-0.033	0.161	-0.144	-0.303	-0.039	0.089	0.296

Values in bold indicate significant correlations (*$P < 0.05$, **$P < 0.01$).

Table S8 Correlation between the relative abundance of fungi functional groups and soil physicochemical variables along the elevational gradients.

Guild groups	Elevation	pH	Moisture	DOC	NO_3^--N	NH_4^+-N	DON	TN	TC	C/N ratio
Plant Pathogen	0.263	0.061	**0.573****	0.166	**0.553****	0.273	0.205	0.264	0.304	0.149
Mycoparasite	0.299	0.028	-0.348	-0.311	-0.311	0.157	0.001	-0.148	-0.271	**-0.405***
Ectomycorrhizal	0.23	-0.015	0.097	-0.121	0.007	**0.578****	0.197	0.241	0.054	-0.338
Foliar Epiphyte	**0.551****	0.285	-0.156	**-0.475***	0.209	-0.091	**-0.499***	**-0.597****	**-0.600****	**-0.427***

Values in bold indicate significant correlations (*$P < 0.05$, **$P < 0.01$).

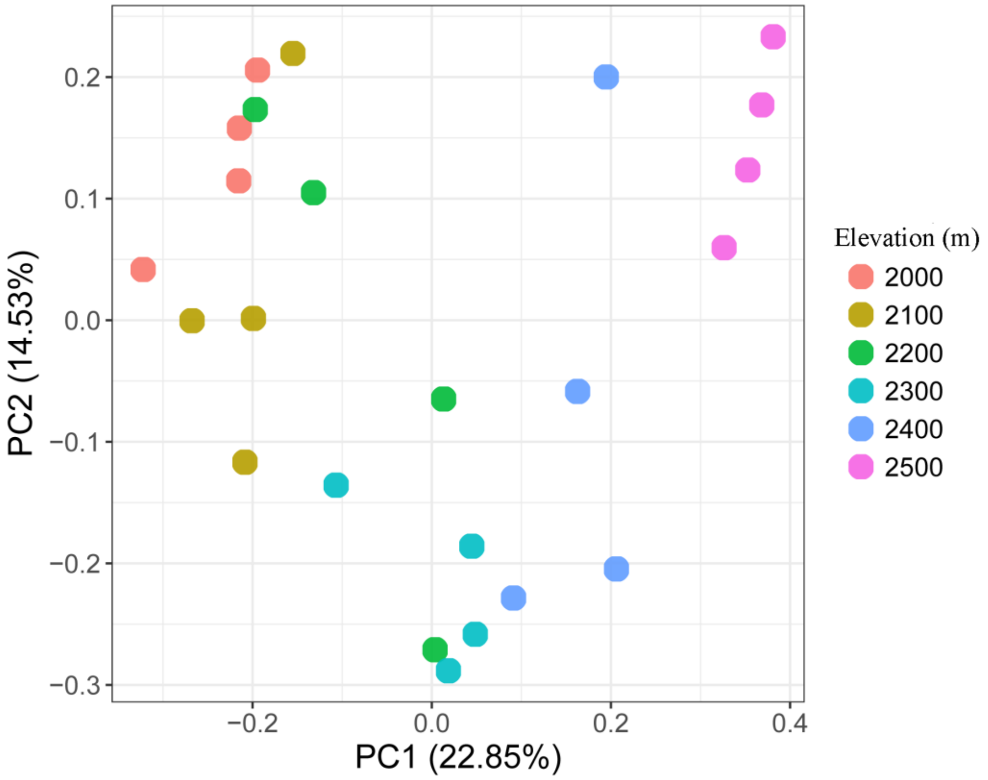

Figure S1 Principal co-ordinates analysis (PCoA) plot depicts soil fungal community composition at different elevations.

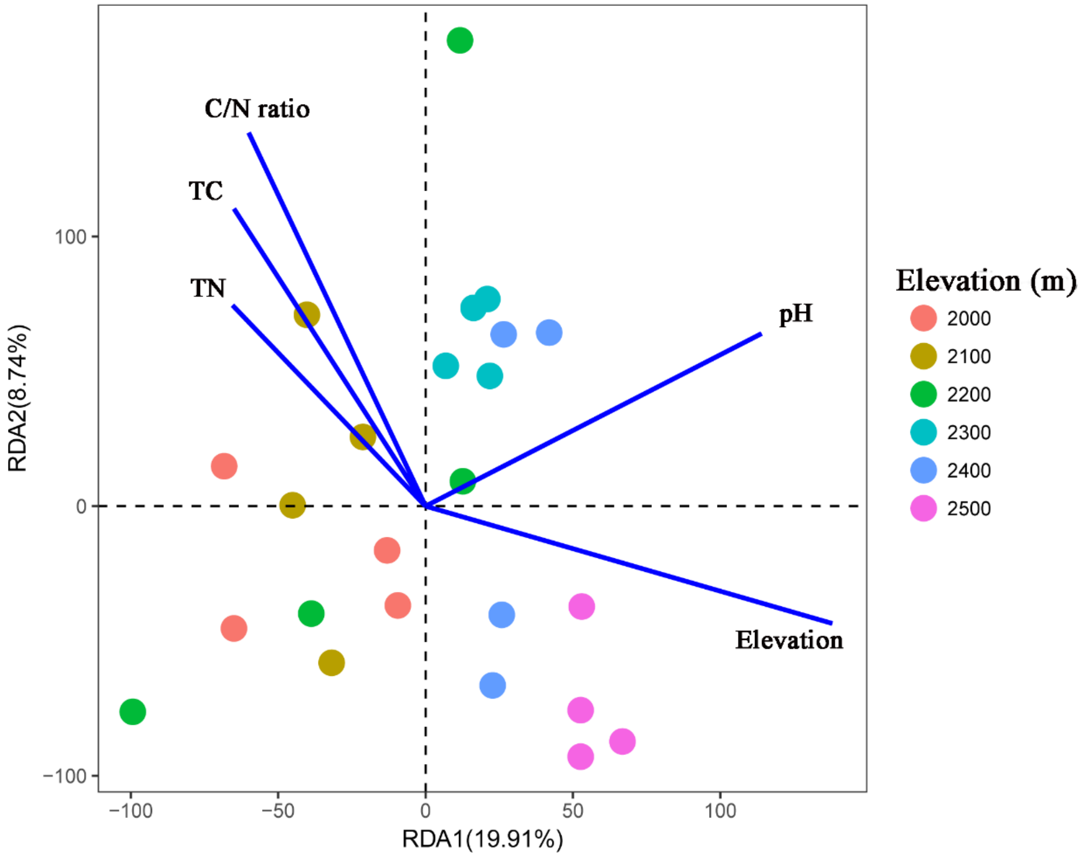

Figure S2 Redundancy analysis (RDA) plot depicting the correlation between fungal communities and major physicochemical variables. TC: total carbon; TN: total nitrogen.
